# Metabolomics-Based Liquid Biopsy for Predicting Clinically Significant Prostate Cancer

**DOI:** 10.3390/cancers17233815

**Published:** 2025-11-28

**Authors:** Yuan-Chi Lin, Chung-Hsin Chen, Ming-Shyue Lee, Cheng-Fan Lee, Pei-Wen Hsiao, Hsiang-Po Huang, Yeong-Shiau Pu

**Affiliations:** 1 Department of Internal Medicine, National Taiwan University Hospital and College of Medicine, Taipei 100225, Taiwan; linyuanchi0@gmail.com; 2 Department of Urology and Institute of Clinical Medicine, National Taiwan University College of Medicine and Hospital, Taipei 100233, Taiwan; duoncin.dan@gmail.com; 3 Department of Biochemistry and Molecular Biology, College of Medicine, National Taiwan University, Taipei 100233, Taiwan; mslee2006@ntu.edu.tw; 4 Department of Biochemistry and Molecular Cell Biology, School of Medicine, College of Medicine, Taipei Medical University, Taipei 11031, Taiwan; bryancflee@tmu.edu.tw; 5 Agricultural Biotechnology Research Center, Academia Sinica, Nankang, Taipei 11529, Taiwan; pwhsiao@gate.sinica.edu.tw; 6 Graduate Institute of Medical Genomics and Proteomics, College of Medicine, National Taiwan University, Taipei 100233, Taiwan

**Keywords:** metabolomics, clinically significant prostate cancer, Gleason score, mass spectrometry, nuclear magnetic resonance, National Comprehensive Cancer Network risk grouping

## Abstract

Prostate cancer (PC) remains a major cause of cancer deaths in men. The serum biomarker prostate-specific antigen (PSA) lacks specificity in distinguishing clinically significant PC (sPC) from insignificant PC (isPC), leading to overdiagnosis and overtreatment. Although magnetic resonance imaging (MRI) improves detection, it is expensive, is time-consuming, and may involve inter-reader discrepancies. Recently, metabolomics, which has a high analytical sensitivity and broad molecular-feature coverage, has emerged as a promising tool to risk-stratify PC. This review examined studies of blood and urine metabolomics for sPC biomarker identification. Significant metabolite changes in sPC patients often involved fatty acid metabolism, sphingolipid metabolism, glycolysis, the citric acid cycle, purine/pyrimidine metabolism, and tyrosine/phenylalanine metabolism. Specifically, more than one study reported increased lactate and phenylalanine levels, along with decreased tyrosine, xanthine, and histidine levels, in sPC patients. Several metabolic panels outperformed serum PSA in predicting sPC, particularly when combined with clinical factors. Among these, two urine-based tests may have higher accuracy in predicting sPC than most current commercially available assays. However, direct comparison between studies may be inappropriate due to methodological heterogeneity, the variability in biospecimen types, inconsistent use of digital rectal examinations, and different sPC definitions and predictive endpoints. Most relevant studies were of small sample size or lacked external validation. Despite these challenges, metabolomics-based liquid biopsies show strong potential for improving sPC detection. Future research should focus on protocol standardization, MRI integration, absolute metabolite quantification, and validation in large and independent cohorts to enhance model credibility.

## Simple Summary

Prostate cancer is one of the most common cancers in men, but not all cases are life-threatening. Current blood tests, like the prostate-specific antigen test, often cannot tell the difference between aggressive and non-aggressive forms of the disease. This can lead to unnecessary treatment. Although magnetic resonance imaging can help, they are expensive and interpretations often vary significantly between radiologists. A promising new approach called metabolomics studies small molecules in blood and urine to identify patterns that distinguish serious prostate cancer from indolent forms. This review examines how these metabolic patterns, including pathways and metabolites consistently reported in more than one study, could assist doctors in better detecting aggressive cancer and avoiding overtreatment. Some of these patterns may even predict aggressive disease more accurately than existing tests. While more research is needed to strengthen these findings, this method could help develop noninvasive tests to support risk assessment before biopsy.

## 1. Introduction

As the most prevalent internal cancer, prostate cancer (PC) is also the second common cause of cancer-related mortality among men in developed countries [1]. Of note, PC is significantly on the rise in developing countries [1]. For clinically indolent or insignificant PC (isPC),usually defined as low Gleason scores (GS), active surveillance or conservative measures remain the treatment of choice [2,3]. Nevertheless, the extensive use of prostate-specific antigen (PSA) testing leads to a high false-positive rate [4,5], and overdetection rates ranging from 27% to 56% [6].

Most PCs detected are asymptomatic and harmless during a man’s lifetime even if left untreated. In contrast, the cost of overtreatment in men with isPC would impair their quality of life, particularly regarding their urinary and sexual function [6]. On the other hand, clinically significant PC (sPC) requires active definitive treatments, either radical resection or radiation therapy, to prevent cancer spread and mortality [7]. Therefore, there is an urgent need for more accurate predictive tools to differentiate sPC from isPC, either before or after prostate biopsy. Accurate tools that predict well “before” biopsy are even more valuable than tools “after” biopsy because the former reduces unnecessary biopsies and overdiagnosis.

As a powerful diagnostic tool, multiparametric MRI (mpMRI) helps ameliorate the rate of unnecessary biopsies [8]. However, mpMRI is costly and time-consuming. A multicenter meta-analysis reported a positive predictive value of only 35%, with substantial variability across centers due to inter-reader discrepancies [9,10]. Extensive omics-based efforts have focused on developing noninvasive predictive models to enhance risk assessment and inform clinical decisions on biopsy.

Recent advances in multi-omics technologies have expanded the biomarker landscape for sPC, encompassing genomics, transcriptomics, proteomics, and increasingly, metabolomics. Commercial liquid biopsy tests, such as PCA3 (urine RNA biomarkers) [11], 4Kscore (blood protein biomarkers) [12], SelectMDx (urine RNA biomarkers) [13], and the Prostate Health Index (serum protein biomarkers) [14], aid in estimating PC or sPC risk, but the diagnostic performance of most remains suboptimal. While genomic and transcriptomic markers offer upstream insights, they may not reflect real-time tumor activity, and RNA-based assays are costly and technically complex due to RNA instability. In contrast, metabolomics captures the end-products of cellular activity, offering a real-time snapshot of tumor physiology and microenvironmental alterations [15]. Given that metabolic reprogramming is a hallmark of aggressive PC [16], metabolomics provides a potentially more sensitive and complementary approach to risk stratification of PC. This rationale underpins our focus on metabolomics-based liquid biopsy as a predictive tool for sPC.

Advances in chromatography and mass spectrometry have driven major progress in this domain. Ultra-high-performance liquid chromatography (UPLC) provides higher resolution and faster separation, while integrated platforms such as liquid chromatography–mass spectrometry (LC-MS) and gas chromatography–mass spectrometry (GC-MS) enable broad metabolite detection [17,18], GC-MS remains strong for low-molecular-weight volatiles, though derivatization is needed for non-volatiles [19], whereas LC-MS is favored for its versatility across polar and non-polar metabolites [20,21,22].

Nuclear magnetic resonance (NMR) spectroscopy, which is based on the same magnetic resonance principles used in MRI, is another major platform, determining metabolite structures through radiofrequency excitation and Fourier transformation of magnetic resonance signals [23]. Proton (^1^H)-NMR is widely used for fluids and optimized with water suppression to detect low-abundance metabolites [24]. Compared with MS-based methods, NMR is non-destructive, highly reproducible, and requires minimal sample preparation, although signal deconvolution is laborious and its analytical sensitivity is lower [25,26].

Urine and blood are the most commonly analyzed samples in metabolomics. Blood samples display good reproducibility owing to the physiological homeostasis of blood. In contrast, the results of urine sample tests may vary greatly because of differences in terms of the subjects’ hydration status or renal concentration ability, both of which can amplify subtle metabolic changes [23]. Blood samples are rich in proteins and lipid compounds, carrying many metabolic end-products and modifications, which can complicate analysis. Conversely, urine contains far fewer proteins due to glomerular filtration, thereby offering a better resolution for NMR analysis and minimizing the number of deproteinization steps for GC-MS or LC-MS [27]. However, the high-water content of urine leads to low levels of lipid metabolites. Notably, due to the high variability of urine samples, normalization is required, either to both urine creatinine and the total useful signals (the sum of metabolite peak areas in the readout [28]) or to osmolality alone [29].

## 2. Research Design and Characteristics

The research approach used for this systematic review was aligned with methods reported elsewhere [30].

Databases and search strategies

We performed a comprehensive literature search of PubMed and Embase from their inception to 30 June 2025. Two investigators independently screened full-text articles for eligibility. The search strategy incorporated the following terms: (Prostate cancer) AND (Clinically significant OR Aggressive OR High-risk OR High-grade) AND (Metabolomics OR Metabolites OR Metabolomic) AND (Blood OR Serum OR Plasma OR Urine OR Semen). We also manually reviewed the reference lists of eligible studies to include or exclude any additional relevant publications.

Study eligibility criteria

A. Inclusion Criteria
  1. Studies involving patients with biopsy-confirmed PC.
  2. Body-fluid samples collected within one year before or after the confirmatory biopsy.
  3. Studies employing metabolomic methods to analyze body-fluid samples.
  4. Articles that defined clinically significant versus insignificant PC or used comparable terminology.
B. Exclusion Criteria
  1. Articles without full-text availability or not written in English.
  2. Studies lacking a clear definition of isPC versus sPC.
  3. Studies without a direct comparison between isPC and sPC (e.g., those only comparing isPC vs. benign and sPC vs. benign separately).
  4. Studies using body-fluid samples collected more than one year before PC diagnosis (e.g., large epidemiological cohorts).
  5. Studies comparing multiple stages of PC without post hoc subgroup analysis.

**Figure 1:**
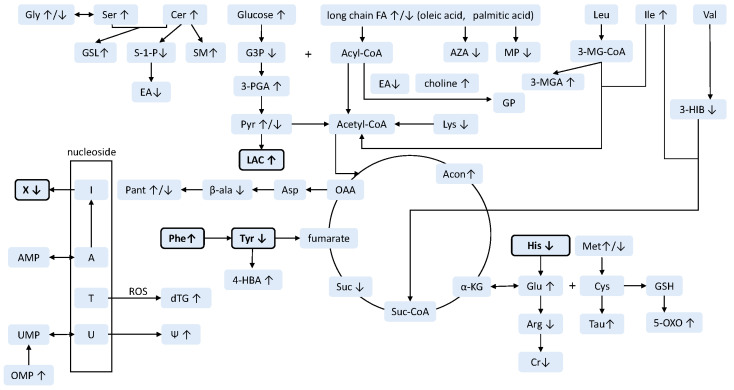
Hypothetical metabolic pathways altered in sPC according to metabolomics studies of blood or urine. This figure maps the differential metabolites between sPC and isPC identified in this review onto known biochemical pathways, under the assumption that blood or urine metabolite changes reflect intracellular metabolic alterations. Bold characters and rectangles indicate dysregulated metabolites reproducibly reported in more than one study. A: adenine, Acon: Aconitate, AMP: adenosine monophosphate, Arg: arginine, Asp: aspartate, AZA: azelaic acid, Cer: ceramide, Cr: creatine, Cys: cysteine, dTG: thymidine glycol, EA: Ethanolamine, FA: fatty acid, Gly: glycine, Glu: glutamate, GP: glycerophospholipid, GSH: glutathione, GSL: glycosphingolipids, G3P: Glycerol 3-phosphate, His: histidine, I: inosine, Ile: isoleucine, LAC: lactate, LCFA: long chain fatty acid, Leu: leucine, Lys: lysine, Met: methionine, MP: monopalmitin, OA: oleic acid, OMP: orotidylic acid, PA: palmitic acid, Pant: pantothenate, Phe: phenylalanine, Pyr: pyruvate, Ser: serine, SM: sphingomyelin, Suc: succinate, S-1-P: sphingosine-1-phosphate, T: thymidine, Tau: taurine, Tyr: tyrosine, U: uridine, UMP: uridine monophosphate, Val: valine, Xant: xanthine, α-KG: α-ketoglutarate, β-ala: β-alanine, Ψ: pseudouridine, 3-HIB: 3-hydroxyisobutyric acid, 3-HIB: 3-hydroxyisobutyric acid, 3-MGA: 3-methylglutaconic acid, 3-MG-CoA: 3-methylglutaconyl-CoA, 3-PGA: 3-Phosphoglyceric acid/glyceric acid, 4-HBA: 4-hydroxybenzoic acid, 5-OXO: 5-Oxoproline. ↑: up-regulation, ↓: down-regulation.

A total of 12 studies were included, most of which successfully identified metabolites or metabolite panels capable of distinguishing sPC from isPC, with varying predicting performance attributable to different study designs and methodologies. Table 1, which summarizes studies using NMR, and Table 2, which summarizes those using MS, present sample sizes, analytic platforms, metabolomic approaches, and statistical methods that have been used in these studies. Table 3 shows the alterations of individual metabolites, either up- or down-regulated, and classifies them into corresponding pathways using the web-based platform MetaboAnalyst 6.0 “http://www.metaboanalyst.ca (accessed on 19 October 2025)”. The hypothetical metabolic pathways perturbed in sPC relative to isPC are illustrated in Figure 1.

## 3. Definition of sPC and isPC Across Different Studies

The studies incorporated in this review predominantly define sPC based on the Gleason grading system, which sums the primary and secondary histological patterns (Gleason grades 1–5) into the GS of 2–10 [44]. Seven studies identified PC with a GS ≥ 7 as sPC [31,32,33,35,36,38,39]. Among them, one study used two different definitions to distinguish isPC from sPC: one based on Gleason score (GS 5 versus GS 7), and the other based on disease extent (organ-confined versus non-organ-confined) [31]. However, a minimum of GS 6—rather than GS 5—should be assigned in prostatectomy specimens, as recommended by the International Society of Urological Pathology (ISUP) Consensus Conference [45]. Moreover, it is well recognized that PC with a predominant pattern of Gleason grade 4 (4 + 3) carries a higher risk of mortality than PC with a predominant pattern of 3 (3 + 4), yet both result in a GS of 7. Notably, patients with GS 7 (3 + 4) who have <50% positive biopsy cores and no additional NCCN intermediate-risk factors (clinical stage T2b–T2c or PSA 10–20 ng/mL) may still be eligible for active surveillance [46]. Thus, relying solely on GS without distinguishing GS 4 + 3 from 3 + 4 may lead to mis-stratification of the PC risk [47]. Some studies have noted this finding. Accordingly, one study adopted the five-tier Gleason Grade Group (GGG) system recommended by the ISUP, categorizing GG1 as low-grade and GG2-5 as intermediate/high-grade PC for comparison [41]. Another study categorized PC by combining both the ISUP GGG system [45] and clinical staging (cT) (or TNM system) [48], classifying PC into the low aggressive group (GG 1 and stage cT1–cT2 and PSA < 10 ng/mL), aggressive group (GG 4 or 5, or PSA > 20 ng/mL, or GG 2-3 plus stage cT3-cT4) and intermediate aggressive group (all others) [37]. Three other studies in this review adopted the National Comprehensive Cancer Network (NCCN) risk groupings [46] to categorize PC as very low risk, low risk, favorable intermediate risk, unfavorable intermediate risk, high risk, and very high risk [38,39,40]. In this review, we did not include studies that only analyzed metabolic changes across more than two clinical stages (e.g., from early, advanced, to metastatic PC) [49] or across multiple Gleason categories [50]. Although these studies reported statistically significant metabolic changes with PC progression, they did not conduct post hoc pairwise comparisons between categories and therefore offered only indirect evidence of risk prediction. Due to the heterogeneity of sPC definitions, however, caution should be exercised when comparing the included studies and assessing the utility of metabolites as predictive markers.

## 4. Methodological Characteristics of the Selected Studies

In most of the studies included in this review, samples were collected after PC diagnosis; only four studies collected samples prior to biopsy [35,40,41,42]. Another methodological characteristic was the frequent use of univariable models, chosen for their simplicity. However, one study lacked methods for multiple-comparison correction, thereby leaving the determined biomarkers at risk of type 1 error [36]. Additionally, one tissue- and serum-based study with the aim of correlating metabolites with the GS of PC reported that no metabolite reached statistical significance after Benjamini–Hochberg false discovery rate (FDR) correction [39].

In multivariable models, feature selection remained challenging, requiring a balance between achieving a high performance and still avoiding overfitting. Logistic regression was widely applied and often combined with a stepwise variable reduction algorithm to construct multivariable models. Two studies utilized the forward stepwise selection algorithm based on the Akaike information criteria to optimize biomarker selection and enhance model robustness [40,42].

Machine learning algorithms have gained a competitive edge in biomarker selection. Among them, partial least squares discriminant analysis (PLS-DA), integrated with the variable importance in projection (VIP) score, has become the most widely recognized method for visualizing group separation and directly identifying predictors. In this review, three studies employed PLS-DA with VIP scoring [37,38], while one utilized the PLS-DA method alone [34]. Furthermore, a refined variant of PLS-DA, orthogonal partial least squares discriminant analysis (OPLS-DA), was applied in two studies [32,33], whereas another study used both PLS-DA and OPLS-DA [35]. Fan et al. implemented the random forest algorithm for model construction, and critical metabolic features were ranked according to the mean reduction in the Gini index [31]. Notably, Penney et al. applied an automated machine learning framework that tested multiple methods (Least Absolute Shrinkage and Selection Operator [LASSO], ridge regression, support vector classifier, random forest), optimized hyperparameters via Bayesian optimization, and combined the best models through ensemble selection [39]. While such approaches improve dimensional reduction and performance, they also carry a high risk of overfitting, especially without external validation.

## 5. Metabolites Differentiating sPC from isPC and Their Potential Mechanisms

The differential metabolites between sPC and isPC, as identified via NMR spectroscopy and MS, are listed in Table 1 and Table 2, respectively, along with the corresponding methodology and study design. The trends in metabolite alterations in sPC are listed in Table 3, categorized by metabolic pathways. Among all alterations, many metabolites can be categorized under fatty acid metabolism, sphingolipid metabolism, the citric acid cycle, glycolysis, purine/pyrimidine metabolism, and phenylalanine/tyrosine metabolism (Table 3). Across these twelve studies, only lactate and phenylalanine were consistently elevated in body fluids, while urinary xanthine and plasma histidine were reproducibly decreased in sPC patients (Table 3). The possible major metabolic pathway alterations are illustrated in Figure 1 and discussed in detail below.

### 5.1. Lipid Metabolism

Sphingolipids are essential components of cell membranes and pivotal mediators of intracellular signal transduction and, together with their related enzymes, are intimately associated with human cancers dissemination [51]. Four out of twelve studies included in this review reported alterations in sphingolipid metabolism during PC progression [32,37,40,42], such as increased levels of ceramides and sphingomyelins [37]. Ceramides can be converted into glycosphingolipids and sphingomyelin via the actions of glucosylceramide synthase and sphingomyelin synthase, thereby potentially enabling tumor cells to evade apoptosis and contributing to drug resistance and increased aggressiveness [37]. Although ceramide-derived sphingosine-1-phosphate (S1P) has been linked to PC formation and progression [52], Nunes et al. [43] reported that patients with isPC exhibited higher plasma S1P levels than those with sPC. They suggested that this might be due to the reduced activity of sphingosine kinase-1 (SphK1) in erythrocytes, potentially secondary to anemia in sPC or other sPC-related factors [43].

Regarding the fatty acid oxidation pathway, carnitine shuttles long-chain fatty acids into mitochondria for β-oxidation via carnitine palmitoyltransferase-1, thereby generating adenosine triphosphate to meet the high energy demands of PC cells [53]. Our current review indicates that among all metabolic categories, fatty acid metabolism harbors the greatest number of differential compounds discriminating sPC (Table 3) [33,40,42]. Interestingly, elevated levels of the short-chain fatty acid acetate [33,42] and decreased levels of the saturated long-chain fatty acid stearic acid [40] have been observed. These findings are consistent with previous reports that enhanced β-oxidation in PC accelerates the stepwise shortening of long-chain fatty acids, ultimately generating acetyl-CoA to meet the high energy demands of tumor growth [53,54]. One may argue that the levels of another saturated long-chain fatty acid, palmitic acid, indeed increased in urine; however, this is in line with a previous case–control study linking higher circulating palmitate and lower stearate levels with PC risk [55]. Mechanistically, palmitate is the primary product of fatty acid synthase, which is often overexpressed in PC, leading to increased palmitate levels in tumor cells [55]. In contrast, stearate can directly inhibit tumor growth through DNA damage and apoptosis [56], suggesting that the level of stearate may be restricted by tumor cells to avoid its cytotoxic effects. Notably, our review extends these findings that this palmitate–stearate shift is evident when comparing isPC and sPC, suggesting its role in both PC initiation and progression.

### 5.2. Carbohydrate Metabolism

The shift from citrate secretion to citrate oxidation is widely recognized as a hallmark of PC cells [57], and this trend was found, through this review, during PC progression. Several studies reported dysregulated metabolites in the citric acid cycle [33,40,42]; reductions in the citric acid cycle metabolites may reflect increased uptake and utilization by tumors [58]. Increased levels of aconitic acid [40], an intermediate of the citric acid cycle, in sPC might be related to a disruption in zinc-mediated inhibition of aconitase in PC cells [59].

Regarding amino sugars, the concurrent elevation in plasma levels of glycoprotein-bound N-acetylglucosamine [34,40] and N-acetylgalactosamine [34,40] in sPC patients suggests activation of the hexosamine biosynthetic pathway, whereas increased levels of plasma glycoprotein-bound N-acetylneuraminic acid [34,40] indicate upregulation of the sialic acid pathway. Together, these changes highlight enhanced glycosylation and sialylation in aggressive tumors, with excess intermediates spilling into urine. This is in agreement with reports showing that O-linked β-N-acetylglucosaminylation levels in PC tissues were correlated with a higher GS and a poorer prognosis [60] and that increased levels of α2-3-linked sialic acid on serum glycoproteins in PC patients can enhance the specificity and sensitivity in predicting GS when compared with PSA [61]. Mechanistically, enhanced glycosylation and sialylation contribute to cancer progression by affecting multiple oncogenic signaling, immune evasion, and metastasis pathways [62,63].

### 5.3. Amino Acid Metabolism

Serine supports the interconnected methionine and folate cycles by donating a one-carbon unit during its conversion to glycine—a reaction catalyzed by serine hydroxymethyltransferase (SHMT). One study found higher blood serine levels in patients with PC with higher GS [32], consistent with the upregulation of multiple enzymes involved in serine biosynthesis in advanced PC tissues [64]. Moreover, higher serum glycine levels were also detected in patients with PC with GS ≥ 7 [33]. The increase in serum glycine might have resulted from upregulated SHMT activity, because it was found based on transcriptomic data that its gene expression was elevated in high-grade PC tissues [33]. In contrast, one study reported significantly decreased urine glycine levels in sPC [40]. Whether this discrepancy was due to increasing renal glycine reabsorption caused by sPC or other factors (e.g., ethnicity, as the latter two studies featured Chinese patient samples) remains to be determined. It is also likely that the concurrent increase in serine and glycine levels reflects increased flux through the serine–glycine–one-carbon pathway, driven by upregulated de novo serine biosynthesis, SHMT activity, and amino acid transporter in PC [65].

Regarding phenylalanine and tyrosine metabolism, the reported increase in phenylalanine [33,40] and decrease in tyrosine [35,39] levels in sPC are consistent with a previous report that decreased urinary tyrosine levels were correlated with increasing PC severity [66]. This may arise from either reduced conversion of phenylalanine to tyrosine or enhanced tyrosine utilization by aggressive PC. The former has been reported in ovarian cancer due to impaired phenylalanine-4-hydroxylase activity under oxidative stress and inflammation [67], while the latter reflects rapid protein synthesis in aggressive cancer, supported by evidence of increased tyrosine uptake through membrane transporters in PC cells [68]. The observed increase in 3,4-dihydroxyphenylacetic acid [40] alongside a decrease in 4-hydroxymandelic acid [40] suggests a metabolic shift in sPC, favoring dopamine degradation over norepinephrine/epinephrine catabolism in catecholamine pathway utilization, as 3,4-dihydroxyphenylacetic acid is a dopamine metabolite, whereas 4-hydroxymandelic acid is a product of catecholamine metabolism. Mechanistically, it is possible that sPC rewires aromatic amino acid metabolism to favor dopamine degradation, as dopamine metabolism generates reactive oxygen species that may promote genomic instability and disease progression [69]. Additionally, elevated levels of 4-hydroxybenzoic acid may also suggest enhanced tyrosine catabolism, as 4-hydroxybenzoic acid can be a downstream product of tyrosine through a less well-characterized pathway involving enzymatic conversion of tyrosine to 4-coumarate and subsequent steps yielding 4-hydroxybenzoate. Alternatively, it might also originate from microbial metabolism [70,71].

Histidine is an essential amino acid in humans and one of the least abundant amino acids in the body. In addition to serving as a building block for protein synthesis, histidine catabolism generates glutamate, which fuels energy metabolism and simultaneously provides one-carbon units to the folate cycle. A smaller fraction of histidine is processed into histamine, which plays important roles in regulating inflammation and immune responses [65]. Three studies in our review reported decreased plasma histidine levels in sPC patients [34,35,39], consistent with a decline in serum histidine levels from BPH and early PC to the most advanced stages of PC [49]. Regarding the mechanistic investigation, in a high-fat diet-induced PC mouse model, expression of the histidine decarboxylase-encoding gene and histamine levels in PC tissues were both increased, while treatment with a histamine receptor antagonist reduced inflammation and suppressed tumor growth [72]. Moreover, a study using liver cancer mouse models has reported that enhanced histidine metabolism does not directly promote tumor proliferation; rather, it modulates the tumor microenvironment by suppressing immune cell function, ultimately creating a niche that favors tumor growth [73]. Although histidine and its metabolites have been proposed to regulate redox states, energy metabolism, or epigenetic remodeling, which affect immune cell function and tumor progression [73], the precise regulatory mechanisms remain largely unclear.

### 5.4. Nucleotide Metabolism

In purine metabolism, xanthine and 1-methyxanthine—intermediates of purine catabolism—were found at lower levels in sPC compared to isPC [40,42], likely due to greater reutilization of purine intermediates in aggressive cancers to sustain increased DNA/RNA synthesis associated with higher cell proliferation [60]. Regarding pyrimidine metabolism, higher urinary levels of orotidylic acid were found in GS7 than in GS6 PC [32]. Since orotidylic acid (namely orotidine-5′-monophosphate) is an intermediate in de novo pyrimidine anabolism, elevated levels of this compound in urine may indicate upregulated pyrimidine biosynthetic flux in sPC. Pseudouridine, one of the modified pyrimidines and the most abundant RNA modification found in various RNA classes, is essential for proper folding and structural integrity of RNA molecules [74]. Elevated levels of pseudouridine have been observed in the urine of sPC patients [40]. This is consistent with the idea that aberrant RNA modifications contribute to cancer development and progression [74]. Additionally, urinary thymidine glycol, a marker of DNA oxidation and damage, was significantly elevated in sPC and increased with GS progression [38], in accordance with the reported greater oxidative stress and DNA damage in more aggressive cancers [75].

Regarding nicotinamide adenine dinucleotide (NAD) metabolism, elevated urinary 1-methylnicotinamide was observed in patients with PC with GS ≥ 7 compared to PC with GS < 7 [33]. This metabolite is produced by nicotinamide N-methyltransferase, which diverts nicotinamide from the NAD^+^ salvage pathway and thereby reduces NAD^+^ regeneration [76]. Methylnicotinamide has been shown to promote proliferation, inhibit apoptosis, and enhance oxidative phosphorylation in cancer cells, thereby potentially limiting the shift to glycolysis and supporting the energy demands of high-grade PC [33,77]. The gene set enrichment analysis performed in the same study also showed that high-GS PC was enriched for gene sets associated with oxidative phosphorylation and depleted for those related to glycolysis [33], which was consistent with the effects of methylnicotinamide.

## 6. Performance of Models Predicting for sPC Across Studies

To distinguish high-grade (GS ≥ 7) PC from low-grade (GS 6) PC, Falegan et al. built an NMR-based OPLS-DA model with fair goodness of fit (R^2^ = 0.62) and predictability (Q^2^ = 0.49) and achieved an AUC of 0.98 [32]. Similarly, Kumar et al. [35] built an OPLS-DA model to distinguish PC with GS ≥ 7 from that with GS6, achieving R^2^Y = 0.704, Q^2^ = 0.412, and an overall accuracy of 0.78 with a 25-metabolite panel. Badmos et al. attempted to stratify PC with different GSs using volatile organic compounds and established a LASSO-regularized logistic regression model with an average cross-validated AUC of 0.78 [41]. Several studies also aimed to distinguish advanced-stage PC from localized PC based on the AJCC staging system. For example, Snider et al. demonstrated that PLS-DA models that consisted of three to five sphingolipids for classifying PC aggressiveness achieved AUC values between 0.842 and 0.882, thus outperforming PSA (AUC 0.742) [37]. Moreover, we recently published two studies on the prediction of sPC based on the NCCN groupings. We showed excellent performance in both urinary GC-MS (AUC 0.89–0.95) and LC-MS (AUC 0.88–0.91) analyses, thus potentially reducing a significant number of unnecessary biopsies [40,42]. Additional statistical methods used to evaluate the predictive value of individual metabolites or panels are summarized in Table 1 and Table 2.

## 7. Comparison of Key Metabolite Alterations in PC Tissues and Body Fluids

McDunn et al. reported increased choline and glycine in non–organ-confined relative to organ-confined prostatectomy tissues [78], consistent with the plasma and serum findings in our review [33]. An NMR-based study by Dudka et al. showed higher choline and glutamate but lower methionine in sPC tissues [79]. The choline and glutamate findings aligned with our plasma and urine data [38], whereas methionine levels in body fluids remained inconsistent [39,42]. Another study found that high-grade PC tissues showed elevated serine but reduced ethanolamine [80], which matched the semen and urine findings for sPC in our review [32,40], whereas their other observations—elevated lysine and decreased lactate—were opposite to the trends in body fluids [32,35,40]. The study by Penney et al., which was also included in our review, found higher fructose in PC tissues from GS ≥ 8 vs. GS = 6 cancers, though fructose tended to decrease in sPC serum without statistical significance [39]. Shao et al. reported elevated glutamate in locally advanced vs. localized PC tissues [81], consistent with our urine data [38], while their observation that succinate correlated positively with GS differed from our included study [42]. Overall, tissue and body-fluid patterns were consistent for choline, glycine, serine, ethanolamine, and glutamate, but differed for lysine, lactate, fructose, and succinate.

## 8. Discussion

Unlike many metabolomics studies that focused on differentiating benign biopsy from PC, far fewer studies were found in the literature that focused on the distinction between sPC and isPC. The main reason for this may be that detecting subtle metabolic differences between sPC and isPC often requires larger sample sizes, highly sensitive analytical technologies, and more advanced mathematical algorithm for model construction that may exceed the resources available for most researchers. However, because not all PC detected needs to be treated, it remains crucial to develop measures that can distinguish sPC from isPC. Many PCs are indolent and can be safely managed with active surveillance or conservative measures. A recent study by Guercio et al. [82] reported that up to 21% of patients experienced significant decision regret after radical prostatectomy, largely due to urinary incontinence and erectile dysfunction. Advances in non-invasive metabolomic panels for detecting sPC may help improve quality of life by reducing unnecessary prostatectomies and their associated complications. Consequently, there is an unmet clinical need for metabolomic marker panels capable of effectively making this nuanced distinction, especially because the definition of sPC may vary with patient age. Given this unmet need, we applied strict inclusion criteria to focus on studies that directly addressed the sPC versus isPC comparison. While studies that reported metabolomic features associated with differentiating PC from benign pathology or disease progression along the PC journey (e.g., bone metastasis, mortality) were excluded from this review. Some metabolites identified in those studies overlapped with those listed in Table 3 and warrant further investigation [49,50].

### 8.1. Expressed Prostatic Secretion Versus Urine Samples

None of the studies in our review collected urine samples after digital rectal prostatic massage, also known as expressed prostatic secretion [EPS] urine. EPS urine is favored by some researchers [83] because it contains the prostatic fluid which may have directly contacted tumors and thus enriched with tumor-derived substances. However, DRE may cause discomfort and introduce cellular debris into the specimen, thus leading to confounding signals and reduced reproducibility. Other disadvantages include the variability in EPS quantity and composition resulting from differences in practitioners’ massage skills. On the other hand, metabolite profiles from urine samples may also vary greatly between individuals due to factors such as diet, hydration status, exercise, medications, and lifestyle, which may complicate interpretation and biomarker validation. In addition, urine is produced via renal filtration of the blood and thus it is more difficult to attribute observed metabolic changes directly to the prostate.

### 8.2. Gleason Grade Group (GGG) Versus NCCN Risk Grouping

For the prediction endpoint, most studies [31,32,33,35,36,37,38,39,41] in this review used only the GS or GGG to stratify PC risks when evaluating metabolite panels; only three [34,40,42] used the NCCN risk grouping [46]. The GGG system, also known as the ISUP Grade Group system [45], is a histopathological classification based on prostate biopsy specimens. It categorizes tumors from GG 1 (GS ≤ 6) to GG 5 (GS 9–10), with tumors of GG ≥ 2 (GS ≥ 7) usually defined as sPC. This GGG system improves the distinction within intermediate-risk PCs by differentiating those with GS 3 + 4 (GG 2) from more aggressive GS 4 + 3 tumors (GG 3), which have different prognostic implications [45].

However, biopsy procedures may introduce potential sampling bias by under-grading or misclassifying aggressive cancers as low risk ones. For example, GG2 (GS 3 + 4) indicates a minor presence of histological pattern 4 but does not quantify its extent, which allows higher-risk tumors with a greater percentage of pattern 4 to be underestimated [84]. Additionally, treatment decisions often require a holistic consideration of other clinicopathological factors.

The NCCN risk grouping integrates multiple clinical variables—including PSA concentration, clinical tumor stage, Gleason GG and many detailed clinicopathological parameters—to produce a better tool of multifactorial risk assessment that reflects the true tumor’s biological behaviors alongside the disease burden [46]. This integration facilitates more comprehensive risk stratification by subcategorizing patients into very low, low, intermediate (favorable and unfavorable), high, and very high-risk groups. This classification is particularly useful for intermediate-risk groups where clinicians are allowed to tailor managements precisely [46,85]. The NCCN system’s ability to predict biochemical recurrence and progression surpasses that of single-factor models due to its incorporation of a broader clinical context, making it a cornerstone in contemporary PC treatment guidelines [46,86].

### 8.3. The Age Factor in Defining sPC

In major clinical practice guidelines, such as the NCCN [46] and the European Association of Urology [3] guidelines, conservative management can be applied in patients with very-low-risk, low-risk, or favorable intermediate-risk disease. These classifications carry significant clinical implications for treatment planning. Additionally, patients’ life expectancy plays a critical role in helping decision-making, as studies have shown that PC may progress slowly, and the vast majority of men with lower-risk diseases are likely to die from causes other than PC. So, it is reasonable that the older the patients, the more conservative the treatment should be. In men with a relatively short life expectancy, FIR disease can be regarded as isPC as we can expect that the chance of dying of FIR PC is low. Thus, stratifying patients with both risk and life expectancy enables more personalized treatment.

According to the NCCN guideline [46], for patients with very-low-risk or low-risk PC, active surveillance is preferred if the life expectancy is ten years or longer (e.g., ages 60–75), whereas observation or watchful waiting is typically recommended for those with a life expectancy under ten years, as these patients are less likely to benefit from aggressive management or repeated surveillance biopsy. In patients with favorable intermediate-risk (FIR) PC, life expectancy plays a critical role in treatment selection. Observation may still be appropriate for those with an estimated life expectancy of five to ten years (e.g., men over 75 to 80), whereas active treatment—such as radical prostatectomy or radiation therapy—should be more strongly considered alongside active surveillance for those expected to live more than ten years [3,46]. In this review series, only two studies addressed the age or life expectancy issue [40,42], namely by developing distinct metabolite marker panels for predicting sPC in patients with varying life expectancies. Consequently, there is a strong need for biomarkers to stratify intermediate-risk patients by guiding conservative measures for men with favorable factors and appropriate definitive therapy for those with unfavorable ones.

### 8.4. Contribution of Marker Panels and Clinical Factors

Although most studies in this review reported clinical factors, such as age, body mass index, smoking history, PSA, diabetes, and cholesterol as baseline characteristics, these were often treated as confounding factors that may have obscured the identification of metabolic markers and require adjustment to appropriately match cases and controls. In contrast, instead of adjusting for these clinical factors, integrating them into the predictive models may significantly increase the sPC prediction accuracy [40,42]. For example, in the GC-MS-based Models II and III reported by Huang et al. [40], the validation AUCs for predicting sPC were 0.93 and 0.88 for metabolic markers alone and 0.81 and 0.84 for five clinical factors alone (age, serum PSA, family history of PC, prior negative biopsy, and abnormal DRE), respectively, while the combination of the two achieved significantly higher AUCs of 0.95 and 0.93.

In the LC-MS-based Models II and III reported by Chen et al. [42], the performance of metabolite markers alone was slightly inferior to five clinical factors alone. Nevertheless, combining metabolite markers with clinical factors consistently improved performance compared to using either alone, yielding validation AUCs of 0.88 in both models. This suggests that both metabolite markers and clinical factors may significantly enhance the other’s performance, providing a distinct and complementary value in predicting sPC and reinforcing confidence in their future clinical utility.

### 8.5. Racial Differences

Although differences in PC incidence among racial groups are well recognized [87], racial disparities in serum or urine metabolite profiles associated with PC risk have been rarely reported [88]. In terms of race-specific metabolite markers distinguishing sPC from isPC, Snider et al. [37] analyzed metabolomic data separately by race and found twenty-four metabolites associated with PC severity (GG 1 vs. GG 2–5) exclusively in African American men and two metabolites unique to European-American men. In addition, this review series includes several studies confined to particular geographic regions or ethnic groups: two studies using urine samples from ethnic Chinese men in Taiwan [40,42], one study using South African plasma [34], and another using plasma from men in India [35]. In the study conducted in South Africa, although the authors performed a subgroup analysis based on Black, Colored, and White sub-ethnic groups, the differences they observed in metabolites among these subgroups did not reach statistical significance [34]. Likewise, in the study by Badmos et al., although four sub-ethnic groups were comparatively represented, no results were reported on metabolite differences among them [41]. Indeed, without well-designed comparative studies, it is unclear whether the identified metabolites are region- or race-specific. In the era of precision medicine, considering patients’ racial and regional backgrounds is crucial, particularly for diseases such as PC where racial differences can be huge. Therefore, since most previous metabolomic studies comparing sPC and isPC have focused on limited populations, race-specific subgroup analyses and inclusion of broader ethnic groups are essential to identify both universal and population-specific metabolite biomarkers for more effective clinical management of PC.

### 8.6. Blood Versus Urine

The choice between serum and urine as the optimal liquid biopsy remains under debate. Urine has an advantage, as prostate-specific metabolites may be directly secreted into it, with less cellular and protein contamination under normal renal function. Metabolite concentrations are also higher in urine than in plasma due to renal processing and secretion [89,90,91]. Nonetheless, urine stability is a concern because metabolite profiles and concentrations from urine samples may vary greatly between individuals and even within the same individual at different times, influenced by factors such as diet, hydration, exercise, medication, and lifestyle. This complicates interpretation and biomarker validation. Therefore, studies using urine often require urine-specific gravity or urine creatinine for normalization [92,93]. The advantage of blood samples lies in their stability. Additionally, blood samples are more suitable for multi-omic analyses, including genomics, transcriptomics, and proteomics studies, because blood samples provide higher-quality DNA, RNA, and proteins [50] than urine. Derezinski et al. compared the performance of serum and urine biomarkers and indicated that urine biomarkers had better sensitivity, while serum was superior in specificity, with a similar overall accuracy [94].

### 8.7. mpMRI and Its Combined Use with Metabolite Biomarkers

mpMRI is emerging as a new standard for identifying aggressive PC in men with elevated PSA biopsy. The Prostate MR Imaging Study (PROMIS) showed that it could spare 27% of patients with elevated PSA levels from unnecessary biopsies [95]. The NCCN guideline recommends mpMRI as a key step before prostate biopsy in at-risk men, since it has an improved risk stratification and diagnostic accuracy. However, a negative mpMRI does not rule out cancer; thus, additional biomarkers have been suggested to inform biopsy [46]. Despite its high negative predictive value, mpMRI often struggles to detect small but high-risk lesions, and substantial discrepancies in MRI reports have been observed between different radiologists [96]. Ambiguous images categorized as PI-RADS 3 are challenging to interpret and provide limited guidance for further management [96].

MRI–ultrasound fusion biopsy strategies that combined targeted and perilesional biopsies have been evaluated as a way to reduce the complications associated with the higher core numbers required in systematic biopsies and to decrease the detection of isPC. Although this approach avoided diagnosing ~3% of isPC, it still missed ~7% of sPC, raising safety concerns [97]. In contrast, some high-performing serum and urine metabolite panels in our study may help triage patients before biopsy, reducing unnecessary procedures while maintaining high sensitivity for detecting sPC, thereby improving pre-biopsy risk assessment.

Chen et al. proposed using metabolomic testing as an initial step before mpMRI and biopsy, reserving the latter for high-risk patients [42]. There is an urgent need of studies comparing the performance of mpMRI, metabolomic panels, and their combined use in different sequences; this might aid pre-biopsy clinical decision-making and support the practical implementation of metabolomic panels in clinical practice.

## 9. Future Perspectives

### 9.1. The Clinical Application of Metabolite Markers

Identification of predictive biomarkers in patients with elevated PSA levels (4–10 ng/mL) may reduce unnecessary biopsies and associated complications [98,99]. Metabolomics offers a highly sensitive approach for improving pre-biopsy risk stratification in PC. To date, only one metabolite-based commercial kit, Prostarix, has been developed for this purpose. It uses a quantitative LC-MS method to measure four amino acids (sarcosine, alanine, glycine, and glutamic acid) in urine samples collected after a prostate message [100]. However, a study performed by Sroka et al. in 2017 found that there were no significant changes in urine sarcosine concentrations between PC and benign prostatic hyperplasia [101]. Moreover, this test is not FDA-approved. It appears that it is no longer actively marketed, and is absent from recent reviews and guidelines. The limited availability of commercial metabolite tests stems from several factors, notably the fact that most findings were based on small studies and require large, multicenter validation. These tests also require MS or NMR, which is not widely used in routine clinical practice. Moreover, variability between metabolomics instruments and sample batches, along with strong competition from RNA and protein biomarker tests, contribute to the limited availability of metabolite tests on the market.

We propose the following solutions to address the scarcity of metabolite tests. First, multicenter large sample-sized and multi-racial international studies with external validation are essential for robust biomarker profile construction. Second, developing smaller, user-friendly, and affordable targeted metabolomics instruments could enable routine testing. Alternatively, centralized services may be an option for sites that lack complex equipment. Third, frequent machine calibration, standardized protocols, and rigorous quality control are crucial to minimize variability across different platforms. Finally, cost-effectiveness analyses and comparisons with DNA-, RNA-, and protein-based tests will help highlight the advantages of metabolomics-based tests.

In summary, while some studies using metabolomics have shown promise in identifying biomarkers for sPC, most findings to date are based on correlation analyses derived from single cohorts. As such, their applicability to diverse clinical populations remains uncertain.

### 9.2. Metabolite-Based Targeted Therapies

Detailed characterization of the metabolic alterations in sPC has led to the development of several metabolic pathway modulators as potential targets for cancer therapies. Serum ceramide levels are elevated in sPC [37], and its metabolite S1P is associated with resistance to therapeutics in PC [102]. Because the activity of sphingosine kinase, which generates S1P, is also significantly elevated in PC [103], the ceramide–S1P axis has emerged as a promising therapeutic target. Opaganib, a sphingosine kinase 2 (SPHK2) inhibitor, reduced S1P production and was tested in a phase-II trial for metastatic castration-resistant PC (CRPC) in combination with androgen receptor inhibitors, where it showed the potential to overcome enzalutamide resistance [104]. Moreover, statins, which inhibit the rate-limiting enzyme of cholesterol synthesis, 3-hydroxy-3-methylglutaryl–coenzyme A reductase, and PCSK9 inhibitors, which block proprotein convertase subtilisin/kexin type 9–mediated LDL receptor degradation, both lower ceramide and cholesterol levels and have been shown to reduce relapse rates and PC-specific mortality [105,106]. Statins also block androgen receptor synthesis in enzalutamide-resistant PC cell lines, thereby helping to slow tumor progression [104]. While it may seem reasonable to target aberrant metabolic pathways based on the metabolite biomarkers identified in various studies, this strategy assumes that the observed alterations accurately reflect metabolic changes within sPC cells. This necessitates a comparative analysis of target metabolite levels in sPC tissues and body fluids, supported or complemented by functional experiments.

## 10. Conclusions

Our review underscores the crucial role of metabolomics in accurately predicting sPC. These metabolic signatures reflect aberrations across multiple metabolic pathways involved in PC. Based on reported performance metrics, non-targeted NMR using patients’ semen showed the highest AUCs (numerically) [32], followed by GC/Q-TOF MS using urine samples [40]. However, further studies are required to confirm these findings, as direct comparisons between studies of different study settings may not be appropriate. Although diagnostic metabolomic panels showed promise, challenges remain, including small sample sizes, the lack of validation cohorts, the need for targeted approaches for precise quantification, suboptimal feature-selection methods, and insufficient prospective studies with long-term follow-up. Future research should focus on large-scale studies with external validation, absolute metabolite quantification, and comparisons of the diagnostic performance of metabolomics-based tests with MRI or in combination with MRI. Identifying reliable biomarkers that may help clinical decision-making and optimize therapy selection for patients at risk remains a long-standing and consistently pursued goal in the era of precision medicine. Realizing this vision will require clear demonstration of clinical utility and cost-effectiveness to enable the integration of metabolite-based markers into everyday practice.

## Figures and Tables

**Table 1:** NMR-based metabolomics studies differentiating sPC from isPC.

Study Groups (*n*, Number of Patients)	Sample Type	Analytic Platform	Metabolomics Approach	Statistical Analysis/ Feature Selection	Results	Year/Reference
14 BPH, 23 GS = 5, 24 GS = 7	serum	gel electrophoresis, NMR	non-targeted	ANOVA, Random forest	Validation (+): internal only; The performance of NMR-detected metabolites was inferior to that of proteomics biomarkers identified by gel electrophoresis	2011/[31]
41 GS = 6, 9 GS = 7, 13 HC	Semen	NMR	non-targeted	Student *t*-test, OPLS-DA (VIP score)	No validation; GS 6 vs. GS 7 model R^2^:0.62, Q^2^:0.49, AUC 0.98 Lysine, serine, pyruvate, and histidine differentiated GS 6 from GS 7 (*p* < 0.05, VIP > 0.75)	2021/[32]
41 GS < 7, 32 GS ≥ 7	Serum/urine	NMR	Targeted	Mann–Whitney U test, OPLS-DA	No validation; OPLS-DA model: R^2^Y = 0.227/0.556 (serum/urine) Q^2^Y = 0.0231/−0.256 (serum/urine) Serum glucose, glycine, and urine methylnicotinamide increased in high GS PC (*p* = 0.015, 0.0369, and 0.0056, respectively)	2022/[33]
NCCN classification 14 very low, low, or intermediate risk, 7 high, 12 very high risk	plasma	NMR	non-targeted	Kruskal–Wallis test with False discovery rate control, Kernel-based feature extraction, Hierarchical clustering	validation (+): internal only; Very high-risk PC was characterized by high levels of GlycA and GlycB (*p* = 0.0186 and 0.0369, respectively)	2021/[34]
15 GS < 7, 35 GS ≥ 7	Plasma	NMR	targeted	Mann–Whitney U test, OPLS-DA (VIP score), Random forest	Validation (+): internal only; In PC with GS ≥ 7, levels of choline, phosphocreatine, lactate, and taurine elevated (AUC: 0.63–0.8), while histidine and tyrosine decreased (AUC: 0.66–0.76), compared to PC with GS < 7	2024/[35]

GS: Gleason score, BPH: benign prostatic hyperplasia, Gly A: N-acetylglucosamine/N-acetylgalactosamine–containing glycoprotein carbohydrates, Gly B: N-acetylneuraminic acid–containing glycoprotein carbohydrates, HC: healthy control, OPLS-DA: Orthogonal Partial Least Squares Discriminant Analysis.

**Table 2:** Mass spectrometry-based metabolomics studies differentiating sPC from isPC.

Study Groups (n, Number of Patients)	Sample Type	Analytic Platform	Metabolomic Approach	Statistical Analysis/ Feature Selection	Results	Year/Reference
49 BPH, 49 PC (GS < 7 vs. ≥7)	urine	LC-MS/MS	targeted	Mann–Whitney U test, ROC analysis	No validation; 3-hydroxyisobutyric acid decreased in GS ≥ 7 PC.; AUC: 0.670, *p* = 0.0391	2018/[36]
57 low aggressive PC, 65 intermediate PC, 37 highly aggressive PC	plasma	UPLC-MS	non-targeted	t-test with False Discovery Rate control, ANOVA, PLS-DA (VIP score)	No validation; 3–5 sphingolipids were most associated with PC aggressiveness AUC: 0.842–0.882	2020/[37]
40 PC (10 samples each for GS 6 to 9), 10 HC	urine	Paper spray ionization-MS	non-targeted	PLS-DA (VIP score)	validation (−); thymidine glycol correlated with PC advancement	2021/[38]
124 PC tissues 105 normal tissues	serum, tissue	LC-MS/MS, GC-MS	non-targeted	Wilcoxon signed rank test, machine learning: greedy ensemble construction (Logistic, LASSO, Ridge Regression, Support Vector Classifier, Random Forest), Bayesian hyperparameter optimization	No validation; 25 metabolites differentiated high GS tumors (≥8) from low GS tumors (6) with unadjusted *p* < 0.05; none passed multiple testing. The machine learning model classified high vs. low GS tumor: AUC: 0.67	2021/[39]
606 PC, 54 mPC 268 benign	urine	GC-TOF-MS	non-targeted	Stepwise logistic regression with Akaike information criterion	External validation; 22–26 biomarkers + 5 clinical factors. sPC prediction Sens: 90%, Spec: 57–87% AUC: 0.89–0.95 Accuracy: 74–88%	2023/[40]
247 PC (139 low grade, 108 intermediate/high grade), 139 controls	urine	GC-MS	non-targeted	PLS-DA (VIP score) logistic regression with LASSO (L1 penalty)	Validation (+): internal only; The model distinguished low from intermediate/high-grade PC. Sens: 0.8, Spec: 0.7, AUC 0.78,	2024/[41]
481 PC, 44 mPC 122 benign	urine	LC-MS	non-targeted	Stepwise logistic regression with Akaike information criterion	External validation; 25–28 biomarkers + 5 clinical factors. sPC prediction Sens: 90%, Spec: 54–65% AUC: 0.88–0.91 Accuracy: 69–77%	2024/[42]

BPH: benign prostatic hyperplasia, GS: Gleason score, GC: gas chromatography. LC: liquid chromatography, LASSO: Least Absolute Shrinkage and Selection Operator, mPC: metastatic PC, Sens.: sensitivity, Spec.: specificity, PLS-DA: Partial Least Squares Discriminant Analysis, VIP: variable importance in projection, TOF: time-of-flight.

**Table 3:** Metabolite alterations in sPC by different metabolic pathways.

Major Metabolic Pathway	Specific Metabolites	Significant Alterations of Metabolomic Signatures in Clinically Significant PC
Lipid	Sphingolipid	serine ↑ [32], ceramides ↑ [37], glycosphingolipids ↑ [37], sphingomyelins ↑ [37], ethanolamine ↓ [40], hydantoin-propionate ↓ [42], sphingosine-1-phosphate ↓ [43]
	Fatty acid	myristoylglycine * ↑ [38], docosahexaenoate (22:6n3) * ↑ [39], long chain fatty acid (palmitic acid ↑ [40], hexadecanoic acid ↓ [42], stearic acid ↓ [40], 10-hydroxyoctadeca-12,15-dienoic acid ↑ [42]), monoethanolamide of undecylenic acid ↑ [42], N-undecanoylglycine ↑ [42], O-adipoyl-L-carnitine ↑ [42], undecylenic acid ↑ [42], azelaic acid ↓ [42], hept-2-enedioic acid ↓ [42]
	Glycerophospholipid	eicosapentaenoylcholine * ↓ [38], choline ↑ [35], 2-Linoleoylglycerophosphoethanolamine * ↓ [39], 1-Oleoylglycerophosphoethanolamine * ↓ [39]
	Glycerolipid	Glycerol-2-phosphate * ↓ [39], Glycerol-3-phosphate * ↓ [39], 1-stearoyl-rac-glycerol ↓ [40], monopalmitin ↓ [40]
Carbohydrate	Citric acid cycle	succinate ↓ [42], aconitic acid ↑ [40], cyclohexylsuccinate (radioplex) ↑ [42], salicylsulfuric acid ↓ [42], hippurate ↓ [42]
	Glycolysis	glucose ↑ [33], pyruvate (pyruvic acid) ↑ * [32]/ ↓ [40], lactate ↑ [35,40], glyceric acid ↑ [40], di-acetyl-lysine ↓ [42], phosphocreatine ↑ [35]
	Amino sugars	N-acetylglucosamine (glycoprotein-bound) ↑ [34], N-acetylgalactosamine (glycoprotein-bound) ↑ [34], N-acetylneuraminic acid (glycoprotein-bound) ↑ [34]
	Monosaccharides	fructose * ↓ [31], 1,5-anhydro-D-glucitol ↓ [40]
	Disaccharides	lactose ↑ [40]
Amino acid	One carbon	glycine ↑ [33] or ↓ [40], serine ↑ [32], methionine ↓ * [39]/ ↑ [42], s-(d-carboxybutyl)-l-homocysteine ↓ [42]
	Arginine biosynthesis and metabolism	glutamate (glutamic acid) ↑ [38], creatine ↓ [38], arginine * ↓ [39], guanidinoacetic acid ↑ [42],
	Branched chain amino acid biosynthesis & catabolism	3-hydroxyisobutyric acid ↓ [36], 3-methylglutaconic acid * ↑ [38], isoleucine ↑ [40],
	Glutathione	gamma-glutamylmethionine * ↓ [39], pyroglutamine * ↓ [39], 5-oxo-D-proline ↑ [42], L-pyroglutamic acid ↓ [40]
	N-acetyl amino acids	4-acetamidobutyric acid ↑ [40]
	Beta-alanine	alpha-alanine * ↓ [39], beta-alanine ↓ [40], pantothenic acid (pantothenate) ↑ [40]/↓ [42]
	Tyrosine and phenylalanine	L-phenylalanine ↑ [33] * [40], tyrosine ↓ [35,39] *, 3,4-dihydroxyphenylacetic acid ↑ [40], 4-hydroxymandelic acid ↓ [40], 4-hydroxybenzoic acid ↑ [40], 2-hydroxyhippurate * ↑ [39]
	Tryptophan	tryptophan * ↓ [39], indoleacetate * ↑ [39], indolepropionate (Indole-3-propionic acid) * ↑ [39]
	Amino acid sulfation	l-tyrosine methyl ester 4-sulfate ↑ [42]
	Other amino acids	lysine ↓ [32], histidine ↓ [34,35,39]/ ↑ * [32], taurine ↑ [35], N-methylproline * ↑ [39], threonine * ↓ [39], L-phenylephrine ↓ [41], acetylcarnosine ↓ [42],
Nucleotide	Nicotinate and nicotinamide	1-methylnicotinamide ↑ [33]
	Purine/Pyrimidine	thymidine glycol ↑ [38], pseudouridine ↑ [40], xanthine ↓ [40,42], 1-methylxanthine ↓ [42], orotidylic acid * ↑ [38]
Peptide		lysylthreonyllysine ↑ [42], 1,3-diazepane-2,4-dione ↑ [42], alanylglycine ↑ [42],
Steroids		cortisone * ↑ [39], pregnenolone sulfate * ↑ [39], dihydrotestosterone sulfate ↓ [42]
Bile acids		glycocholenate sulfate * ↓ [39], taurolithocholate 3-sulfate * ↓ [39]
Organic acid	Sulfur-containing dicarboxylic acid	3,3′-thiodipropanoate ↑ [42]
Others	Bacterial metabolism	galacturonic acid ↓ [40], quinic acid ↓ [40], 5-hydroxyindole ↑ [40], d-allose ↓ [40], arabinofuranose ↑ [40], l-arabinitol ↑ [40]
	Xenobiotics	trimethylamine N-oxide * ↓ [38], quinone/1,2-benzoquinone ↓ [38], methyl glyphosate ↑ [41], semicarbazide ↑ [41], 2-phenyl-3-decyn-1-ol ↓ [41], n,n-diisopropylethylamine ↓ [41], xylitol ↑ [39] */ ↓ [40], ononitol ↑ [40], galangin ↑ [40], acetamide ↑ [40], 2,5-dipropyltetrahydrofuran ↑ [40], 2,4-dihydroxybutanoic acid ↑ [40], diethanolamine ↓ [40], 3-phenyl-5,10-secocholesta-1(10),2-dien-5-one ↓ [40], tartronic acid ↓ [40], ethyl 1-penten-3-ynesulfonate ↓ [40], levulinic acid ↓ [40], threonic acid ↑ [40], N-(1,4-Dihydroxy-4-methylpentan-2-yl)-3-hydroxy-5-oxo-6-phenylhexanamide ↓ [42], 3-(Cyclobutanecarbonyloxy)-4-(trimethylazaniumyl)butanoate ↑ [42], 1,3,5-tris(2,2-dimethylpropionylamino)benzene ↑ [42], N-butylformamide ↓ [42], dibutyl phthalate ↑ [42], vanilloylglycine ↓ [42], s-(3-oxopropyl)-n-acetylcysteine ↓ [42], 1-methyl-pyrogallol-3-o-sulphate ↓ [42], n-feruloylglycine ↓ [42]
	Industrial chemicals	butane-1,4-diol ↑ [41], 5-ethyl-1,2,3,4-tetrahydronaphthalene ↑ [41]

Up-regulation and down-regulation are shown as ↑ and ↓, respectively. * Tentative changes (not statistically confirmed).

## Data Availability

No new data were created in this review.

## Abbreviations

AUC	Area under the Receiver Operating Characteristic curve
BPH	Benign prostatic hyperplasia
DRE	Digital rectal examination
EPS	Expressed prostatic secretion
FIR	Favorable intermediate risk
GC-MS	Gas chromatography–mass spectrometry
GG	Gleason grade
GGG	Gleason grade group
GS	Gleason score
ISUP	International Society of Urological Pathology
LASSO	Least absolute shrinkage and selection operator
LC-MS	Liquid chromatography–mass spectrometry
mpMRI	Multiparametric MRI
MS	Mass Spectrometry
NAD	Nicotinamide adenine dinucleotide
NCCN	National Comprehensive Cancer Network
NMR	Nuclear magnetic resonance
OPLS-DA	Orthogonal partial least squares discriminant analysis
PC	Prostate cancer
PLS-DA	Partial least squares discriminant analysis
PSA	Prostate-specific antigen
S1P	Sphingosine-1-phosphate
SHMT	Serine hydroxymethyltransferase
sPC	Clinically significant prostate cancer
UPLC	Ultra-high-performance liquid chromatography
VIP	Variable importance in projection
isPC	Insignificant prostate cancer
